# Synthesis of Spirooxindole-*O*-Naphthoquinone-Tetrazolo[1,5-*a*]Pyrimidine Hybrids as Potential Anticancer Agents

**DOI:** 10.3390/molecules23092330

**Published:** 2018-09-12

**Authors:** Liqiang Wu, Yunxia Liu, Yazhen Li

**Affiliations:** 1 School of Pharmacy, Xinxiang Medical University, Xinxiang 453003, China; 2 SanQuan Medical College, Xinxiang Medical University, Xinxiang 453003, China; liuyunxia9477@hotmail.com (Y.L.); liyazhen8478@hotmail.com (Y.L.)

**Keywords:** spirooxindole, *O*-naphthoquinone, hybrids, antitumor activity

## Abstract

A series of novel spirooxindole-*O*-naphthoquinone-tetrazolo[1,5-*a*]pyrimidine hybrids were designed, synthesized and evaluated as potent antitumor agents. These hybrids exhibited relatively high cytotoxic activity against cancer cell line HepG2 (IC_50_ = 2.86–36.34 μM), while normal cell line LO2 was less sensitive to these hybrids (IC_50_ = 36.37–248.39 μM). On the whole, among all the compounds tested, compound **4e**, with a mean IC_50_ value of 2.86 μM, was the most active. The novel hybrids may find their pharmaceutical applications after further investigations.

## 1. Introduction

Multicomponent reactions (MCRs) have become an increasingly favored and useful tool for the synthesis of chemically and biologically important compounds because of their convergence, atom economy, and other suitable characteristics from the point of view of green chemistry [1]. These reactions, which involve at least three different simple substrates, are powerful for the expedient building up of molecular complexity and diversity. In the past decade, there has been tremendous development in three- and four-component reactions, and great efforts continue to be made to develop new multi-component reactions [2].

**Figure 1:**
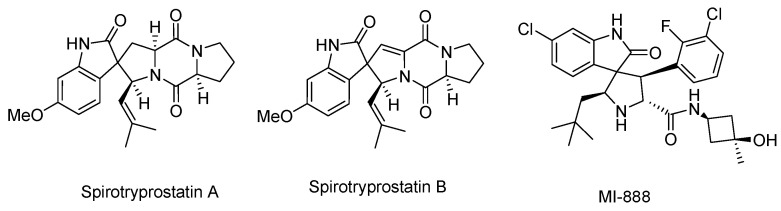
Spirooxindoles with anticancer activities.

Spirooxindole is an important heterocyclic system, which is found in numerous natural products and biologically active molecules. These spirooxindoles seem to be promising candidates for anticancer drug discovery, since they incorporate both oxindoles and other heterocyclic moieties simultaneously. In recent years, the anticancer activity of the spirooxindole has aroused widespread concern, as a number of spirooxindoles have shown different degrees of anticancer activities mainly based on the spiro rings fused at the C3 position of oxindole scaffold and substituents on the oxindole nucleus. Among these, many natural spirooxindoles like spirotryprostatins A and B (Figure 1) also show excellent anticancer activities. More importantly, some synthesized spirooxindoles such as MI-888 (Figure 1) have been in preclinical research for the treatment of human cancers [3].

**Scheme 1:**
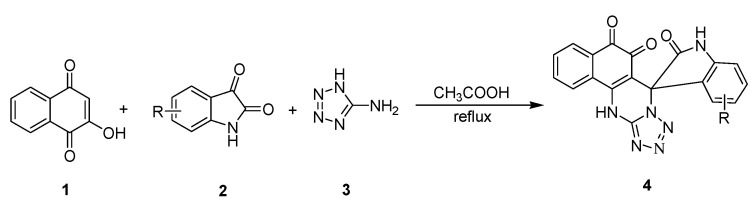
Synthesis of spirooxindole-*O*-naphthoquinone hybrids.

Naphthoquinone core is an active pharmacophore and is used in medicinal chemistry and drug discovery research [4,5,6,7,8]. Recently, some *O*-naphthoquinones, including β-lapachone [9] and tanshinone IIA [10] and dunnione [11], have been reported to kill many human cancers selectively through rapid reactive oxygen species (ROS) generation mediated by NQO1 bioreduction. In view of the limited structure of natural *O*-quinones, it is urgent to develop novel, efficient and diversified *O*-quinones as antitumor agent. In our previous work, we have found that a series of *O*-naphthoquinones possessing tetrazolo[1,5-*a*]pyrimidine scaffolds exhibited significant antitumor activity [12]. There has been significant interest in the combination of two or three pharmacophores on the same scaffold leading to hybrid molecules [13]. These hybrids combine two or three active moieties in a single molecule with the goal of creating a chemical entity that is medically more effective than its individual components. As a result, spirooxindole-*O*-naphthoquinone-tetrazolo[1,5-*a*]pyrimidine, which combine three biologically active heterocyclic cores, are expected to be of pharmacological interest. The one-pot synthesis of these ternary hybrid molecules was achieved through the three-component condensation of 2-hydroxy-1,4-naphthoquinone, isatins, and 5-aminotetrazole in refluxing acetic acid (Scheme 1). These hybrid molecules have been tested in vitro for their antitumor activity against cancer (HepG2) and normal cells (LO2).

## 2. Results and Discussion

To achieve the optimum conditions, the synthesis of 1,6-dihydro–spiro[benzo[*h*]tetrazolo[5,1-*b*]-quinazoline-6,3′-indoline]-2′,7,8-trione (**4a**) was used as a model reaction under a variety of different conditions. The effects of solvents and temperature were evaluated for this reaction, and the results are summarized in Table 1. The exploration of different solvents, such as EtOH, DMF, CH_3_COOH, CH_3_CN, CHCl_3_ and H_2_O, showed that the solvent had an obvious influence on the reaction yield. The best result was obtained in CH_3_COOH. Then, the optimal reaction temperature was further investigated (entries 7–10), and reflux temperature with the highest yield of 49% (entry 3) was chosen as the best suitable condition for all further reactions.

**Figure 2:**
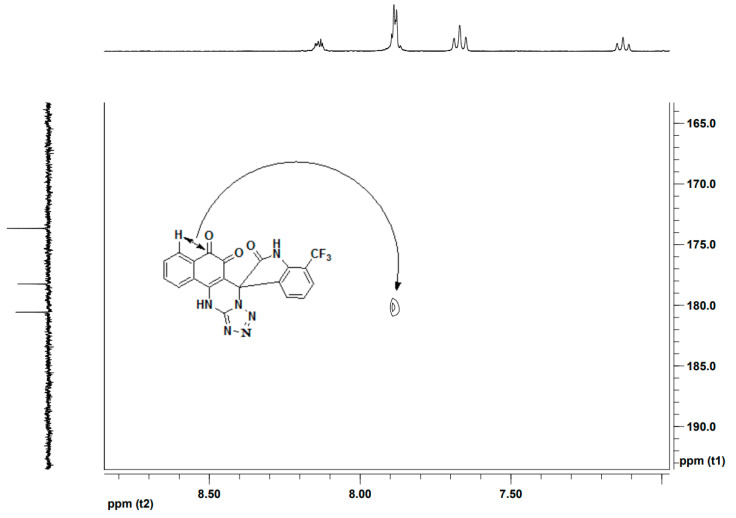
Locally amplified HMBC of **4n**.

Diversity-oriented synthesis is a strategy used by chemical biologists to create a huge diversity of small molecules with potentially useful properties. With optimal conditions in hand, we then carried out the design and diversity-oriented synthesis of novel ternary hybrid molecules. Different isatins were applied to this reaction for the aim to access target molecules with structural diversity. As shown in Table 2, the protocol is amenable to a wide scope of isatins. All of the isatins **2** gave the expected products in moderate yields, whether bearing electron-withdrawing groups (such as halide and nitro) or electron-donating groups (such as an alkyl group), under the same reaction conditions. Therefore, we conclude that the electronic nature of the substitutents of the isatins had no significant effect on the reaction. In all cases the structures of these ternary hybrid molecules were characterized by spectroscopic methods. For example, the high resolution mass spectrum of **4n** displayed the quasi-molecular ion ([M − H]^+^) peak at *m*/*z* = 437.0615, which was consistent with the 1:1:1 adduct of 2-hydroxy-1,4-naphthoquinone, 7-trifluoromethylisatin and 5-aminotetrazole with the loss of two water molecules. The ^1^H-NMR spectrum of **4n** exhibited two singlets due to the NH protons at δ = 12.81 ppm and δ = 11.71 ppm. The H-12 occur as a dt peak at 8.14 ppm, more downfield than expected of aromatic protons. This is explicable by the close proximity of these protons to the lone pairs of the neighboring nitrogen and the consequent anisotropic and van de Waals deshielding. Twenty-three distinct resonances were shown by ^13^C-NMR spectrum of **4n** (the CF_3_ group are quartet due to the ^13^C-^19^F coupling). Among them, four characteristic signals at δ = 60.5 ppm (due to the spiro-C group), 180.2 and 178.2 ppm (arising from the two nonequivalent carbonyl groups), 173.7 (belong to the NH–C=O group) were shown. The HMBC (^1^H detected heteronuclear multiple bond correlation) experiments can observe typical two-bond and three-bond proton to carbon couplings. It could be observed that the long-range correlations of carbonyl carbon C-8 to the protons H-9 of the benzene-fused ring in HMBC spectrum of **4n**, which was able to prove that the formation of the ortho-quinone units in the reaction (Figure 2).

**Scheme 2:**
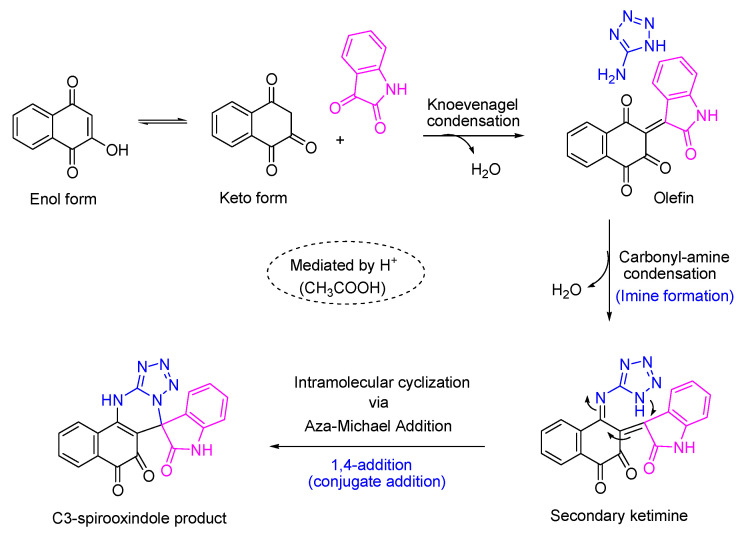
A suggested pathway for the formation of the hybrid.

A suggested pathway for the formation of the hybrid is shown in Scheme 2. Keto form of 2-hydroxy-1,4-naphthoquinone undergoes Knoevenagel condensation with isatin to form olefin. The olefin can react with 5-aminotetrazole by carbonyl-amine condensation to produce the secondary ketamine, which further undergoes intramolecular cyclization via aza-Michael addition to generate C3-spirooxindole product.

Cytotoxicity studies were performed on these ternary hybrid molecules with cell survival being determined by the MTT assay against HepG2 (cancer cell lines) and LO2 (normal hepatic cell lines). Tanshinone IIA (TSA) was used as positive control. As shown in Table 3, most target compounds exhibited relatively higher anticancer activities against HepG2, with IC_50_ values ranging from 2.86–36.34 μM. The results in Table 3 also showed some important structure–activity relationships (SARs) for this series of derivatives. It appears from the data that introduction of 5-F, 5-Me, 7-Cl and 7-CF_3_ group into the spirooxindole group was found to be quite favorable for increasing anti-proliferative activity as seen in compounds **4g**, **4j**, **4f** and **4n** against HepG2 cell line. These results revealed that the position of substituent on the spirooxindole group has a remarkable effect on their anti-proliferative activity. In addition, it could also be noticed that the introduction of small steric hindrance groups (**4g** and **4j**) on indole showed more potent inhibitory effects than large steric hindrance groups (**4e** and **4i**). On the contrary, these compounds possessed relatively poor cytotoxicity against LO2 cells. Compounds with better selectivity towards HepG2 were supposed to have a superior safety profile. It was worth noting that the most selective substrate (**4g**) was potent to the HepG2 cell lines and weakly active to the LO2 cell lines (shifting the IC_50_ 20.6-fold). This compound showed much more safety than the control TSA (shifting the IC_50_ 2.7-fold) in cellular assay.

## 3. Materials and Methods

### 3.1. General Information

NMR spectra were determined on Bruker AV-400 spectrometer (Bruker, Karlsruhe, Germany) at room temperature using tetramethylsilane (TMS) as internal standard. Chemical shifts (*d*) are given in ppm and coupling constants (*J*) in Hz. High resolution mass spectra were recorded on a Bruker micrOTOF-QIII mass spectrometer (Bruker, Villigen, Switzerland). Melting points were determined on a XT-4 binocular microscope (Beijing Tech Instrument Co., Beijing, China) and were uncorrected. Commercially available reagents were used throughout without further purification unless otherwise stated.

### 3.2. General Procedure for the Synthesis of Compound ***4***

To a mixture of 2-hydroxy-1,4-naphthoquinone (1 mmol), isatin (1 mmol) and CH_3_COOH 10 mL, 5-aminotetrazole (1 mmol) was added. The mixture was stirred at reflux for an appropriate time (Table 2). After completion of the reaction (TLC), the reaction mixture was cooled to room temperature and the solvent was evaporated under reduced pressure. Then, the crude product was washed sequentially with 20 mL saturated NaHCO_3_ and 20 mL brine, purified by silica gel column chromatography using petroleum ether: ethyl acetate (*v:v* = 1:1) as eluent to afford the pure product **4** (the copies of all spectral data see Supplementary Materials).

*1,6-Dihydro–spiro[benzo[h]tetrazolo[5,1-b]-quinazoline-6,3′-indoline]-2′,7,8-trione* (**4a**). Orange powder, m.p. 275–277 °C, ^1^H-NMR (400 MHz, DMSO-*d*_6_) δ: 12.65 (s, 1H), 11.18 (s, 1H), 8.12 (m, 1H), 7.87 (dd, 3H, *J* = 6.0, 9.2 Hz), 7.33 (dd, 2H, *J* = 8.4, 16.0 Hz), 7.02 (d, 1H, *J* = 7.6 Hz), 6.92 (t, 1H, *J* = 7.6 Hz); ^13^C-NMR (100 MHz, DMSO-*d*_6_) δ: 180.5, 178.3, 173.1, 149.0, 141.7, 140.7, 135.7, 134.6, 131.6, 131.4, 131.0, 127.0, 126.4, 124.3, 123.9, 115.1, 111.0, 66.1; HRMS-ESI (*m*/*z*): calcd for C_19_H_9_N_6_O_3_ [M − H]^+^: 369.0736, found: 369.0741.

*5′-Bromo-1,6-dihydro–spiro[benzo[h]tetrazolo[5,1-b]-quinazoline-6,3′-indoline]-2′,7,8-trione* (**4b**). Orange powder, m.p. > 300 °C, ^1^H-NMR (400 MHz, DMSO-*d*_6_) δ: 12.70 (s, 1H), 11.33 (s, 1H), 8.13 (m, 1H), 7.89 (d, 3H, *J* = 2.8 Hz), 7.67 (d, 1H, *J* = 1.2 Hz), 7.53 (dd, 1H, *J* = 2.0, 8.4 Hz), 7.00 (d, 1H, *J* = 8.4 Hz); ^13^C-NMR (100 MHz, DMSO-*d*_6_) δ: 180.4, 178.5, 173.0, 149.3, 141.8, 135.7, 134.4, 131.7, 131.6, 131.2, 130.8, 126.9, 126.8, 126.4, 125.4, 112.3, 110.7, 65.6; HRMS-ESI (*m*/*z*): calcd for C_19_H_8_N_6_O_3_Br [M − H]^+^: 446.9847, found: 446.9836.

*5′-Chloro-1,6-dihydro–spiro[benzo[h]tetrazolo[5,1-b]-quinazoline-6,3′-indoline]-2′,7,8-trione* (**4c**). Orange powder, m.p. > 300 °C, ^1^H-NMR (400 MHz, DMSO-*d*_6_) δ: 12.71 (s, 1H), 11.30 (s, 1H), 8.13 (dd, 1H, *J* = 2.0, 6.0 Hz), 7.88 (d, 3H, *J* = 3.2 Hz), 7.54 (d, 1H, *J* = 2.0 Hz), 7.40 (dd, 1H, *J* = 2.4, 8.4 Hz), 7.04 (d, 1H, *J* = 8.4 Hz); ^13^C-NMR (100 MHz, DMSO-*d*_6_) δ: 180.4, 178.5, 173.0, 149.3, 141.8, 135.7, 134.4, 131.7, 131.6, 131.2, 130.8, 126.9, 126.8, 126.4, 125.4, 112.3, 110.7, 65.6; HRMS-ESI (*m*/*z*): calcd for C_19_H_8_ClN_6_O_3_ [M − H]^+^: 403.0352, found: 403.0347.

*6′-Bromo-1,6-dihydro–spiro[benzo[h]tetrazolo[5,1-b]-quinazoline-6,3′-indoline]-2′,7,8-trione* (**4d**). Yellow powder, m.p. 281–283 °C, ^1^H-NMR (400 MHz, DMSO-*d*_6_) δ: 12.72 (s, 1H), 11.35 (s, 1H), 8.14–8.11 (m, 1H), 7.88 (t, 3H, *J* = 2Hz), 7.32 (d, 1H, *J* = 8.0 Hz), 7.19 (d, 1H, *J* = 1.6 Hz), 7.14 (dd, 1H, *J* = 1.6, 8.0 Hz); ^13^C-NMR (100 MHz, DMSO-*d*_6_) δ: 180.4, 178.4, 173.0, 149.3, 144.5, 142.0, 135.7, 134.5, 131.6, 130.8, 129.2, 127.0, 126.9, 126.4, 125.7, 124.1, 113.8, 110.8, 65.3; HRMS-ESI (*m*/*z*): calcd for C_19_H_8_N_6_O_3_Br [M − H]^+^: 446.9847, found: 446.9836.

*1′-Methyl-7′-fluoro-1,6-dihydro–spiro[benzo[h]tetrazolo[5,1-b]-quinazoline-6,3′-indoline]-2′,7,8-trione* (**4e**). Orange powder, m.p. > 300 °C, ^1^H-NMR (400 MHz, DMSO-*d*_6_) δ: 12.84 (s, 1H), 8.13 (dd, 1H, *J* = 3.6, 8.0 Hz), 7.87 (d, 3H, *J* = 2.0 Hz), 7.37 (dd, 1H, *J* = 8.4, 12.0 Hz), 7.28 (d, 1H, *J* = 3.6 Hz), 7.06–7.01 (m, 1H), 3.53 (s, 3H); ^13^C-NMR (100 MHz, DMSO-*d*_6_) δ: 180.5, 178.3, 171.5, 149.2, 147.2 (d, ^1^*J_CF_* = 187.5 Hz), 141.9, 135.7, 134.6, 131.7, 131.6 (d, ^3^*J_CF_* = 9.8 Hz), 130.7 (d, ^2^*J_CF_* = 20.0 Hz), 130.5, 127.0, 126.4, 124.8 (q, ^3^*J_CF_* = 4.6 Hz), 121.3 (d, ^4^*J_CF_* = 2.0 Hz), 119.6 (d, ^2^*J_CF_* = 15.0 Hz), 110.8, 65.0, 29.8; HRMS-ESI (*m*/*z*): calcd for C_20_H_10_FN_6_O_3_ [M − H]^+^: 401.0798, found: 401.0792.

*7′-Chloro-1,6-dihydro–spiro[benzo[h]tetrazolo[5,1-b]-quinazoline-6,3′-indoline]-2′,7,8-trione* (**4f**). Yellow powder, m.p. >> 300 °C, ^1^H-NMR (400 MHz, DMSO-*d*_6_) δ: 12.77 (s, 1H), 11.68 (s, 1H), 8.13 (dd, 1H, *J* = 1.2, 4.4 Hz), 7.89 (d, 3H, *J* = 2.8 Hz), 7.44 (d, 1H, *J* = 8.0 Hz), 7.34 (d, 1H, *J* = 3.6 Hz), 6.97 (t, 1H, *J* = 8.0 Hz); ^13^C-NMR (100 MHz, DMSO-*d*_6_) δ: 180.5, 178.3, 173.1, 149.0, 141.7, 140.7, 135.7, 134.6, 131.6, 1314.4, 130.8, 127.0, 126.4, 124.3, 123.9, 115.1, 111.0, 66.1; HRMS-ESI (*m*/*z*): calcd for C_19_H_8_ClN_6_O_3_ [M − H]^+^: 403.0352, found: 403.0343.

*5′-Fluoro-1,6-dihydro–spiro[benzo[h]tetrazolo[5,1-b]-quinazoline-6,3′-indoline]-2′,7,8-trione* (**4g**). Orange powder, m.p. 297–299 °C, ^1^H-NMR (400 MHz, DMSO-*d*_6_) δ: 12.71 (s, 1H), 11.19 (s, 1H), 8.13 (dd, 1H, *J* = 2.0, 6.0 Hz), 7.88 (d, 3H, *J* = 2.8 Hz), 7.36 (dd, 1H, *J* = 2.4, 8.0 Hz),7.21 (d, 1H, *J* = 2.4 Hz), 7.18 (dd, 1H, *J* = 2.4, 7.2 Hz), 7.02 (dd, 1H, *J* = 4.0, 8.4 Hz); ^13^C-NMR (100 MHz, DMSO-*d*_6_) δ: 180.3, 178.5, 173.2, 158.1 (d, ^1^*J_CF_* = 177.5 Hz), 149.3, 142.1, 139.1, 135.7, 134.4, 131.7, 131.3, 131.1(d, ^3^*J_CF_* = 6.0 Hz), 126.9, 126.4, 117.7 (d, ^2^*J_CF_* = 17.6 Hz), 113.1 (d, ^2^*J_CF_* = 18.8 Hz), 111.7 (d, ^3^*J_CF_* = 5.9 Hz), 110.8, 65.9; HRMS-ESI (*m*/*z*): calcd for C_19_H_8_FN_6_O_3_ [M − H]^+^: 387.0642, found: 387.0633.

*7′-Bromo-1,6-dihydro–spiro[benzo[h]tetrazolo[5,1-b]-quinazoline-6,3′-indoline]-2′,7,8-trione* (**4h**). Orange powder, m.p. 286–289 °C, ^1^H-NMR (400 MHz, DMSO-*d*_6_) δ: 12.76 (s, 1H), 11.54 (s, 1H), 8.13 (m, 1H), 7.89 (s, 3H), 7.56 (dd, 1H, *J* = 0.8, 8.0 Hz), 7.37 (d, 1H, *J* = 7.2 Hz), 6.90 (t, 1H, *J* = 8.0 Hz); ^13^C-NMR (100 MHz, DMSO-*d*_6_) δ: 180.5, 178.3, 173.1, 149.0, 142.4, 135.7, 134.6, 134.3, 131.6, 131.4, 130.7, 127.0, 126.4, 124.7, 124.3, 111.0, 103.2, 66.3; HRMS-ESI (*m*/*z*): calcd for C_19_H_8_N_6_O_3_Br [M − H]^+^: 446.9847, found: 446.9835.

*1′-Phenyl-1,6-dihydro–spiro[benzo[h]tetrazolo[5,1-b]-quinazoline-6,3′-indoline]-2′,7,8-trione* (**4i**). Yellow powder, m.p. 314–317 °C, ^1^H-NMR (400 MHz, DMSO-*d*_6_) δ: 12.84 (s, 1H), 8.16–8.13 (m, 1H), 7.94–7.88 (m, 3H), 7.70 (t, 2H, *J* = 8.0 Hz), 7.60–7.56 (m, 3H), 7.50 (d, 1H, *J* = 6.8 Hz), 7.42–7.37 (m, 1H), 7.07 (t, 1H, *J* = 7.6 Hz), 6.91 (d, 1H, *J* = 8 Hz); ^13^C-NMR (100 MHz, DMSO-*d*_6_) δ: 180.8, 178.3, 171.3, 149.3, 143.9, 141.8, 135.7, 134.6, 134.3, 131.7, 131.6, 130.9, 130.6, 129.3, 127.0, 126.5, 125.5, 124.4, 111.3, 110.2, 65.4; HRMS-ESI (*m*/*z*): calcd for C_25_H_13_N_6_O_3_[M − H]^+^: 445.1049, found: 445.1044.

*5′-Methyl-1,6-dihydro–spiro[benzo[h]tetrazolo[5,1-b]-quinazoline-6,3′-indoline]-2′,7,8-trione* (**4j**). Orange powder, m.p. 267–269 °C, ^1^H-NMR (400 MHz, DMSO-*d*_6_) δ: 12.63 (s, 1H), 11.06 (s, 1H), 8.14–8.11 (m, 1H), 7.87 (dd, 3H, *J* = 5.6, 8.8Hz), 7.14 (d, 2H, *J* = 9.2 Hz), 6.91 (d, 1H, *J* = 8.0 Hz), 2.13 (s, 3H); ^13^C-NMR (100 MHz, DMSO-*d*_6_) δ: 180.4, 178.4, 173.1, 149.1, 141.5, 140.3, 135.7, 134.4, 132.1, 131.6, 131.5, 130.8, 130.1, 126.9, 126.4, 125.5, 111.5, 110.6, 65.8, 20.9; HRMS-ESI (*m*/*z*): calcd for C_20_H_11_N_6_O_3_ [M − H]^+^: 383.0893, found: 383.0889.

*6′-Chloro-1,6-dihydro–spiro[benzo[h]tetrazolo[5,1-b]-quinazoline-6,3′-indoline]-2′,7,8-trione* (**4k**). Orange powder, m.p. 291–293 °C, ^1^H-NMR (400 MHz, DMSO-*d*_6_) δ: 12.63 (s, 1H), 11.06 (s, 1H), 8.14–8.11 (m, 1H), 7.87 (d, 3H, *J* = 3.2 Hz), 7.14 (d, 2H, *J* = 8.6 Hz), 6.91(d, 1H, *J* = 8.0 Hz); ^13^C-NMR (100 MHz, DMSO-*d*_6_) δ: 180.4, 178.5, 173.2, 149.3, 144.4, 142.1, 135.7, 135.6, 134.5, 131.7, 130.8, 128.4, 126.9, 126.7, 126.4, 122.7, 111.0, 110.8, 65.2; HRMS-ESI (*m*/*z*): calcd for C_19_H_8_ClN_6_O_3_ [M − H]^+^: 403.0352, found: 403.0338.

*6′-Methoxyl-1,6-dihydro–spiro[benzo[h]tetrazolo[5,1-b]-quinazoline-6,3′-indoline]-2′,7,8-trione* (**4l**). Orange powder, m.p. 281–283 °C, ^1^H-NMR (400 MHz, DMSO-*d*_6_) δ: 12.58 (s, 1H), 11.11 (s, 1H), 8.13–8.11 (m, 1H), 7.87 (t, 3H, *J* = 2.0 Hz), 7.21 (d, 1H, *J* = 8.4 Hz), 6.55 (d, 1H, *J* = 2.0 Hz), 6.44 (dd, 1H, *J* = 2.0, 8.4 Hz), 3.77 (s, 3H); ^13^C-NMR (100 MHz, DMSO-*d*_6_) δ: 180.3, 178.6, 173.7, 161.9, 149.2, 144.2, 135.7, 134.4, 131.7, 130.7, 126.9, 126.3, 126.1, 122.3, 111.4, 107.5, 97.7, 65.6, 55.9; HRMS-ESI (*m*/*z*): calcd for C_20_H_11_N_6_O_4_ [M − H]^+^: 399.0842, found: 399.0845.

*5′-Trifluoromethoxyl-1,6-dihydro–spiro[benzo[h]tetrazolo[5,1-b]-quinazoline-6,3′-indoline]-2′,7,8-trione* (**4m**). Yellow powder, m.p. 273–276 °C, ^1^H-NMR (400 MHz, DMSO-*d*_6_) δ: 12.74 (s, 1H), 11.39 (s, 1H), 8.15–8.13 (m, 1H), 7.88 (d, 3H, *J* = 2.8 Hz), 7.58 (d, 1H, *J* = 1.2Hz), 7.37 (d, 1H, *J* = 8.4 Hz), 7.12 (d, 1H, *J* = 8.4 Hz); ^13^C-NMR (100 MHz, DMSO-*d*_6_) δ: 180.5, 178.4, 173.2, 149.1, 144.1, 142.1, 135.8, 134.5, 132.1, 132.0, 131.6, 131.2, 130.7, 126.4, 125.8 (q, ^1^*J_CF_* = 177.5 Hz), 121.7, 119.2, 111.9, 111.7, 65.6; HRMS-ESI (*m*/*z*): calcd for C_20_H_8_F_3_N_6_O_4_ [M − H]^+^: 453.0559, found: 453.0564.

*7′-Trifluoromethyl-1,6-dihydro–spiro[benzo[h]tetrazolo[5,1-b]-quinazoline-6,3′-indoline]-2′,7,8-trione* (**4n**). Yellow powder, m.p. 288–290 °C, ^1^H-NMR (400 MHz, DMSO-*d*_6_) δ: 12.81 (s, 1H), 11.71 (s, 1H), 8.15–8.13 (m, 1H), 7.88 (t, 3H, *J* = 2.0 Hz), 7.67 (t, 2H, J = 8.0 Hz), 7.13 (t, 1H, *J* = 8.0 Hz); ^13^C-NMR (100 MHz, DMSO-*d*_6_) δ: 180.6, 178.2, 173.7, 149.1, 142.0, 140.3, 135.7, 134.5, 131.5, 131.4, 129.3, 127.9, 127.0, 126.6 (q, ^1^*J_CF_* = 202.5 Hz), 126.5, 123.3, 112.0, 111.7, 110.8, 64.5; HRMS-ESI (*m*/*z*): calcd for r C_20_H_8_F_3_N_6_O_4_ [M − H]^+^: 437.0610, found: 437.0615.

### 3.3. Anti-Proliferative Assay

HepG2 and LO2 cells were maintained in DMEM (Gibco, Invitrogen Corporation, New York, NY, USA) medium supplemented with 10% FBS, streptomycin (100 μg/mL) and penicillin (100 units/mL) and incubated at 37 °C, 5% CO_2_. HepG2 cells (300,000 cells/ML) and LO2 cells (300,000 cells/ML) were seeded into 96-well plates and incubated at 37 °C in 5% CO_2_/95% air condition. Serially two-fold diluted test compound solutions of each drug were added 48 h later, and the cells were incubated for the next 48 h. The final concentrations of compounds in the sample wells ranged from 1 μM to 1000 μM. After 72 h, 20 mL 3-(4,5-dimethylthiazol-2-yl)-2,5-diphenyl tetrazolium bromide (MTT, 5 mg/mL) was added to each well and the cells were incubated for an additional 4 h. Then, 100 μL, DMSO (dimethyl sulfoxide) were added into each well for dissolving the intracellular formazan crystals. Optical density at 570 nm of each plate was measured with a tunable microplate reader. Each group was in triplicate samples and each drug was divided into at least 5 concentrations. The percentage of absorbance from the sample-treated cells compared to that of the vehicle control (treated with DMSO) was calculated. The resulting cytotoxic acitvities were expressed as IC_50_ values and IC_50_ values were determined using analysis software (Excel) (2013, Microsoft, Redmond, WA, USA).

## 4. Conclusions

In summary, a series of spirooxindole-*O*-naphthoquinone-tetrazolo[1,5-*a*]pyrimidine hybrids was synthesized via the one-pot three-component reaction of -hydroxy-1,4-naphthoquinone, isatins, and 5-aminotetrazole. These hybrids represent a promising class of cytotoxic agents with potential novel therapeutic values. These findings encourage further investigations around this interesting antitumor chemotype.

## Figures and Tables

**Table 1:** Reaction conditions optimization for the synthesis **4a**.

Entry	Solvent	Temperature/°C	Time/h	Yield/% ^1^
1	EtOH	reflux	10	20
2	DMF	120	6	29
3	CH_3_COOH	reflux	6	49
4	CH_3_CN	reflux	10	11
5	CHCl_3_	reflux	10	trace
6	H_2_O	reflux	10	trace
7	CH_3_COOH	25	24	-
8	CH_3_COOH	50	24	trace
9	CH_3_COOH	100	10	22
10	CH_3_COOH	110	7	29

^1^ Isolated yield.

**Table 2:** Preparation of compounds **4.**

Entry	R	Time/h	Product	m.p./°C	Yield/%
1	H	6	**4a**	275–277	49
2	5-Br	8	**4b**	>300	31
3	5-Cl	8	**4c**	>300	32
4	6-Br	8	**4d**	281–283	35
5	1-CH_3_-7-F	5	**4e**	>300	52
6	7-Cl	5	**4f**	>300	61
7	5-F	5	**4g**	297–299	69
8	7-Br	5	**4h**	286–289	57
9	1-C_6_H_5_	6	**4i**	>300	55
10	5-CH_3_	6	**4j**	267–269	48
10	6-Cl	7	**4k**	291–293	45
12	6-OCH_3_	7	**4l**	281–283	46
13	5-OCF_3_	8	**4m**	273–276	36
14	7-CF_3_	7	**4n**	288–290	47

**Table 3:** Antiproliferative activities of compounds **4.**

Comp.	IC_50_ (μM)		Selectivity Ratio ^1^
	HepG2	LO2	
**4a**	23.63	40.15	1.7
**4b**	23.41	65.29	2.79
**4c**	21.93	53.03	2.42
**4d**	36.34	248.39	6.84
**4e**	31.83	54	1.70
**4f**	3.03	49.47	16.33
**4g**	2.86	58.92	20.60
**4h**	20.74	82.34	3.97
**4i**	27.87	75.57	2.71
**4j**	12.58	36.37	2.89
**4k**	21.19	51.99	2.45
**4l**	17.29	78.6	4.55
**4m**	18.29	48.42	2.65
**4n**	7.9	49.9	6.32
**TSA**	23.85	65.29	2.74

^1^ IC_50_ (LO2)/IC_50_ (HepG2).

## Supplementary Materials

The following are available online. ^1^H-NMR, ^13^C-NMR and HMRS of compound **4.**
