# Correction: Wu et al. Synthesis of Spirooxindole-*O*-Naphthoquinone-Tetrazolo[1,5-*a*]Pyrimidine Hybrids as Potential Anticancer Agents. *Molecules* 2018, *23*, 2330

**DOI:** 10.3390/molecules29225257

**Published:** 2024-11-07

**Authors:** Liqiang Wu, Yunxia Liu, Yazhen Li

**Affiliations:** 1 School of Pharmacy, Xinxiang Medical University, Xinxiang 453003, China; 2 SanQuan Medical College, Xinxiang Medical University, Xinxiang 453003, China; liuyunxia9477@hotmail.com (Y.L.); liyazhen8478@hotmail.com (Y.L.)

In the original publication [1], we found that there was a mistake in the structures of compound **4**. As the figure below shows, the structure of compound **4** should be “1,6-dihydro” instead of “4,6-dihydro” [2,3]. The authors did not notice the mistake before the article was published, and they would like to thank Dr. Essam M. Eliwa who pointed it out. A correction is needed for all data describing the structure of compound **4**. The corrections of the figures, schemes, and data affected appear below.

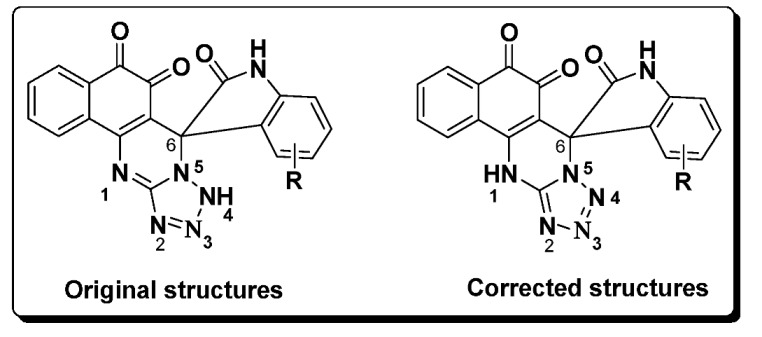



## Graphical Abstract

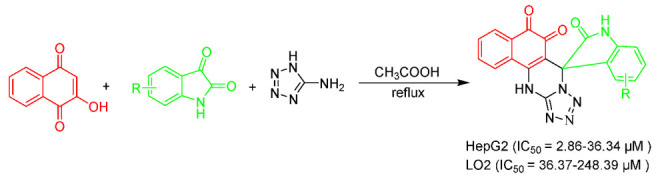



## Error in Results and Discussion

In the original publication [1], there was a mistake in both the first and third paragraphs of the Results and Discussion section. The correct text appears below.

To achieve the optimum conditions, the synthesis of 1,6-dihydro–spiro[benzo[*h*]tetrazolo[5,1-*b*]-quinazoline-6,3′-indoline]-2′,7,8-trione (**4a**) was used as a model reaction under a variety of different conditions.

A suggested pathway for the formation of the hybrid is shown in Scheme 2. Keto form of 2-hydroxy-1,4-naphthoquinone undergoes Knoevenagel condensation with isatin to form olefin. The olefin can react with 5-aminotetrazole by carbonyl-amine condensation to produce the secondary ketamine, which further undergoes intramolecular cyclization via aza-Michael addition to generate C3-spirooxindole product.

## Error in Schemes and Figure

The corrected Scheme 1 appears below:

The corrected Figure 2 appears below:

The corrected Scheme 2 appears below:

## Error in Materials and Methods

In the original publication [1], there was a mistake in the compound names described in Section 3.2. of the Materials and Methods. The correct compound names appear below.

*1,6-Dihydro–spiro[benzo[h]tetrazolo[5,1-b]-quinazoline-6,3′-indoline]-2′,7,8-trione* (**4a**)
*5′-Bromo-1,6-dihydro–spiro[benzo[h]tetrazolo[5,1-b]-quinazoline-6,3′-indoline]-2′,7,8-trione* (**4b**)
*5′-Chloro-1,6-dihydro–spiro[benzo[h]tetrazolo[5,1-b]-quinazoline-6,3′-indoline]-2′,7,8-trione* (**4c**)
*6′-Bromo-1,6-dihydro–spiro[benzo[h]tetrazolo[5,1-b]-quinazoline-6,3′-indoline]-2′,7,8-trione* (**4d**)
*1′-Methyl-7′-fluoro-1,6-dihydro–spiro[benzo[h]tetrazolo[5,1-b]-quinazoline-6,3′-indoline]-2′,7,8-trione* (**4e**)
*7′-Chloro-1,6-dihydro–spiro[benzo[h]tetrazolo[5,1-b]-quinazoline-6,3′-indoline]-2′,7,8-trione* (**4f**)
*5′-Fluoro-1,6-dihydro–spiro[benzo[h]tetrazolo[5,1-b]-quinazoline-6,3′-indoline]-2′,7,8-trione* (**4g**)
*7′-Bromo-1,6-dihydro–spiro[benzo[h]tetrazolo[5,1-b]-quinazoline-6,3′-indoline]-2′,7,8-trione* (**4h**)
*1′-Phenyl-1,6-dihydro–spiro[benzo[h]tetrazolo[5,1-b]-quinazoline-6,3′-indoline]-2′,7,8-trione* (**4i**)
*5′-Methyl-1,6-dihydro–spiro[benzo[h]tetrazolo[5,1-b]-quinazoline-6,3′-indoline]-2′,7,8-trione* (**4j**)
*6′-Chloro-1,6-dihydro–spiro[benzo[h]tetrazolo[5,1-b]-quinazoline-6,3′-indoline]-2′,7,8-trione* (**4k**)
*6′-Methoxyl-1,6-dihydro–spiro[benzo[h]tetrazolo[5,1-b]-quinazoline-6,3′-indoline]-2′,7,8-trione* (**4l**)
*5′-Trifluoromethoxyl-1,6-dihydro–spiro[benzo[h]tetrazolo[5,1-b]-quinazoline-6,3′-indoline]-2′,7,8-trione* (**4m**)
*7′-Trifluoromethyl-1,6-dihydro–spiro[benzo[h]tetrazolo[5,1-b]-quinazoline-6,3′-indoline]-2′,7,8-trione* (**4n**)

In addition, the original text was also corrected to address minor text formatting issues.

## Error in Supplementary Materials

The structures of the above-mentioned compounds **4a**–**4n** were corrected in the ^1^H-NMR, ^13^C-NMR and HRMS data in the Supplementary Materials. 

The authors state that the scientific conclusions are unaffected. The original publication has also been updated.

## Figures and Tables

**Scheme 1:**
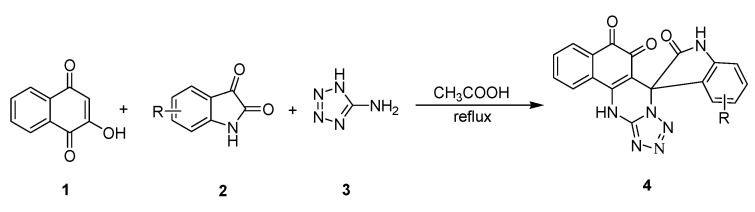
Synthesis of spirooxindole-*O*-naphthoquinone hybrids.

**Figure 2:**
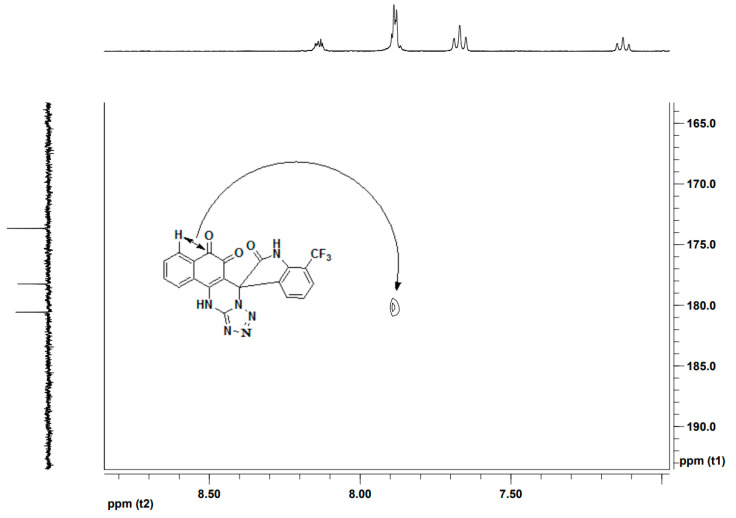
Locally amplified HMBC of **4n**.

**Scheme 2:**
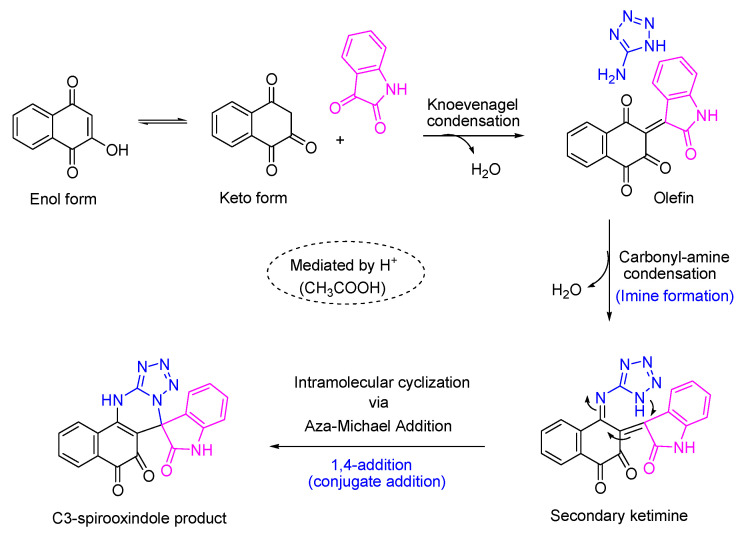
A suggested pathway for the formation of the hybrid.
